# Therapeutic Effects of Polyphenols on the Treatment of Colorectal Cancer by Regulating Wnt *β*-Catenin Signaling Pathway

**DOI:** 10.1155/2021/3619510

**Published:** 2021-09-28

**Authors:** Mehran Pashirzad, Thomas P. Johnston, Amirhossein Sahebkar

**Affiliations:** ^1^Department of Medical Biochemistry, School of Medicine, Mashhad University of Medical Sciences, Mashhad, Iran; ^2^Division of Pharmacology and Pharmaceutical Sciences, School of Pharmacy, University of Missouri-Kansas City, Kansas City, MO, USA; ^3^Biotechnology Research Center, Pharmaceutical Technology Institute, Mashhad University of Medical Sciences, Mashhad, Iran; ^4^Applied Biomedical Research Center, Mashhad University of Medical Sciences, Mashhad, Iran; ^5^School of Pharmacy, Mashhad University of Medical Sciences, Mashhad, Iran

## Abstract

Colorectal cancer (CRC) is the third most common cause of cancer-related death worldwide in terms of both its rates of incidence and mortality. Due to serious side effects associated with conventional chemotherapeutic treatments, many natural products with fewer adverse side effects have been considered as potential treatment options. In fact, many natural products have widely been used in various phases of clinical trials for CRC, as well as in *in vitro* and *in vivo* preclinical studies. Curcumin (CUR) and resveratrol (RES) are classified as natural polyphenolic compounds that have been demonstrated to have anticancer activity against CRC and are associated with minimal side effects. By regulating select target genes involved in several key signaling pathways in CRC, in particular, the Wnt *β*-catenin signaling cascade, the course of CRC may be positively altered. In the current review, we focused on the therapeutic effects of CUR and RES in CRC as they pertain to modulation of the Wnt *β*-catenin signaling pathway.

## 1. Introduction

Colorectal cancer (CRC) is the third most common cause of cancer-related death worldwide in terms of both its rates of incidence and mortality, which are increasing every year [1]. There are various conventional therapies for the treatment of patients with CRC, which include surgery, radiotherapy, and chemotherapy. All of these treatment modalities have severe side effects such as drug toxicity, drug resistance, and hepatic injury [2, 3]. Thus, in view of the increased side effects with conventional treatment methods, there has been increased interest to identify novel and effective therapies with fewer side effects for treating patients with CRC [4]. Based on recent findings concerning CRC treatment, many natural compounds have been extensively studied and used in various phases of clinical trials, as well as in *in vitro* and *in vivo* preclinical studies [5, 6].

Curcumin (CUR) is a natural product derived from the rhizome of *Curcuma longa* with proven efficacy to treat a number of diseases and exhibiting minimal side effects [7, 8]. This polyphenolic compound exhibits numerous pharmacological properties against a variety of pathological conditions [9–17]. In addition, it has been shown that CUR can inhibit many molecular signaling pathways, in particular, the Wnt *β*-catenin signaling pathway, which is associated with its anticancer effects in a variety of human CRC cell lines [14, 18].

Resveratrol (RES) is also a polyphenolic compound and is extracted from many plants such as grapes, peanuts, and berries [19]. RES has proven chemopreventive properties and can influence the pathogenesis of many diseases though with conflicting results [20–23]. It is known that the therapeutic properties of RES are associated with its anti-inflammatory, antiangiogenic, and antioxidant effects [24, 25]. Recent results of several studies have shown that RES can target several molecular signaling pathways, including the Wnt *β*-catenin signaling pathway, and inhibit cell proliferation and induce apoptosis in many solid tumors such as CRC [25, 26].

Wnt signaling is the most common pathway involved in the regulation of the cell cycle, stemness, and cancer [27]. Wnt signaling represents one of the initial key signaling cascades involved in CRC recurrence. Moreover, there exists a strong relationship between the inhibition of this pathway and the exposure of cancer cells to CUR and RES [28, 29]. In this review, we summarize the underlying mechanisms of the antitumor activity of CUR and RES via inhibition of the Wnt *β*-catenin signaling pathway in tumorigenesis and the recurrence of CRC.

## 2. Wnt *β*-Catenin Signaling Pathway

The Wnt signaling pathway can generally be divided into canonical Wnt *β*-catenin signaling and noncanonical Wnt signaling [30]. In the present review, the role of Wnt *β*-catenin signaling in CRC is discussed. The regulation of cellular processes induced by Wnt *β*-catenin signaling is associated with the presence, or absence, of Wnt ligands, which are known to be secreted glycoproteins [31]. In the absence of Wnt proteins, the *β*-catenin destruction complex is derived from intracellular molecular signals including adenomatous polyposis coli (APC), Axin (a scaffolding protein controlling *β*-catenin stability), glycogen synthase kinase 3*β* (GSK3*β*), and casein kinase 1*α* (CK1*α*) [32]. Following the formation of the *β*-catenin destruction complex, phosphorylation and ubiquitination of *β*-catenin are mediated by GSK3*β* and *β*-transducin repeat-containing protein (*β*-TrCP), with eventual translocation of *β*-catenin into the cell nucleus and inhibition in the expression of target genes involved in regulating cellular processes [33]. In contrast to the inactivation of the Wnt *β*-catenin pathway, canonical Wnt signaling is characterized by binding of Wnt proteins to Frizzled receptors (Fzd) and low-density lipoprotein receptor-related protein 5/6 (LRP5/6), phosphorylation of Disheveled (Dvl), inhibition of the *β*-catenin destruction complex, translocation of *β*-catenin into the nucleus, and *β*-catenin and T-cell factor/lymphocyte enhancer factor (TCF/LEF) induced overexpression of target genes such as cyclin D1 and c-Myc, respectively [34].

In a state of homeostasis, Wnt *β*-catenin signaling pathway plays an important role in regulating the cell cycle, as well as the growth, proliferation, and differentiation of cells [35]. In contrast to the homeostatic state, aberrant activation of canonical Wnt *β*-catenin signaling induced by genetic/epigenetic disorders promotes proliferation and tumor growth of cancer cells, in particular, CRC cells [36]. The dysregulation in Wnt *β*-catenin signaling can be inhibited by antibodies [37], shRNA molecules [38], Wnt signaling inhibitors [39], and an impressive number of natural compounds during CRC progression [40].

### 2.1. Wnt *β*-Catenin Signaling and CRC

Epithelial cells of the colon and small intestine normally comprise the functional unit termed the crypt, which includes the crypt base, transit-amplifying cells, and terminally differentiated cells [41]. The crypt base region contains different cells, including Paneth cells and crypt base columnar cells (CBCs). Their cellular processes include proliferation, differentiation, and apoptosis, which are significantly dependent on the activation of Wnt *β*-catenin transduction [42]. In contrast, there are a wide range of genetic and epigenetic changes involved in the progression of CRC, which are closely associated with mutations and dysregulation of the Wnt *β*-catenin signaling components, such as APC and *β*-catenin proteins located at the crypt base of the colonic epithelium [43]. Although Wnt *β*-catenin signaling is considered the initial pathway involved in the early stages of CRC, it also plays an important role in regulating gene expression and several cellular processes. For example, these cellular processes include invasion, proliferation, migration, differentiation, and apoptosis from the time of initiation to the time of recurrence of CRC [44]. On the other hand, it has been determined that the development of APC and *β*-catenin mutation-induced overactivation of Wnt *β*-catenin signaling also occurs in the early stages of CRC [45]. Along these lines, mutation and dysregulation of APC trigger the accumulation of cytoplasmic *β*-catenin, translocation of the *β*-catenin into the nucleus, and overexpression of genes associated with the progression of CRC [45]. An increase in the expression levels of genes of the Wnt *β*-catenin cascade involved in CRC, which include c-Myc [46], Cyclin D1 [47], matrix metalloproteins (MMPs) [48], and Musashi1 (Msi1) [49], results in the activation of aberrant Wnt *β*-catenin signaling in colonic epithelial cells and subsequent development and progression of CRC. Many of the biological and pharmacological factors normally thought of as Wnt antagonists, can affect disturbed regulation of Wnt *β*-catenin signaling, and can be considered as potential therapeutic agents for various types of colon cancer.

### 2.2. Components of Wnt *β*-Catenin Signaling as Therapeutic Targets for CRC

Due to the rate of recurrence of cancer in patients with CRC, therapeutic treatment methods such as biotherapy, radiotherapy, surgery, and curative antibodies in combination with conventional chemotherapy are used [50]. Regarding recent clinical trials targeting different stages of CRC, combination therapy of Wnt antagonists with other therapeutic interventions, which include natural compounds [51], nonsteroidal anti-inflammatory drugs (NSAIDs) [52], and biological inhibitors [53], is currently considered an effective treatment strategy for CRC. Two such natural compounds that can effectively modulate impaired Wnt *β*-catenin signaling are CUR and RES. It has been shown that these natural compounds such as micronized resveratrol (SRT501; NCT00920803) [54], resveratrol-rich fresh red grapes [55], genistein (NCT01985763), quercetin (NCT00003365), and green tea (NCT04345978), in combination with chemotherapeutic agents including celecoxib (NCT00295035) [56], 5-fluorouracil (NCT02724202) [57], and irinotecan (NCT01859858) [58], can effectively target dysregulated components of Wnt *β*-catenin signaling associated with the progression of CRC. The characteristics of various natural compounds, especially CUR and RES (i.e., two natural compounds that are the subject of this review), as they pertain to modulation of the Wnt *β*-catenin signaling at different stages of CRC, are summarized in Table 1. As mentioned above, this review discusses the inhibitory effects of polyphenolic natural products, with the primary focus on CUR and RES, in a wide variety of CRC cell lines.

### 2.3. Antitumor Activity of Curcumin Mediated by Wnt *β*-Catenin Signaling in Different Cellular Processes of CRC Cells

Based on the results of previous studies, curcumin inhibits aberrant activation of several signaling pathways including the Wnt *β*-catenin signaling pathway, which is activated by many tumorigenic factors such as azoxymethane (AOM), dextran sodium sulfate (DSS), and dibenzazepine (DBZ) as determined in various *in vitro* and *in vivo* studies [59, 60]. Regarding recent findings concerning CUR's anticancer activity, this natural product can also inhibit different cellular processes, which include cell proliferation [61], apoptosis [62], cell viability [63], cell cycle dynamics [64], invasiveness [65], and metastasis [66] of various CRC cell lines.

### 2.4. Cell Proliferation

Using a mouse model in which CRC was induced via AOM and DSS, together with real-time quantitative polymerase chain reaction (RT-qPCR) and immunohistochemistry (IHC), Hao et al. have shown that CUR significantly suppresses tumor cell proliferation of colorectal tissue through the downregulation of interleukin-1*β* (IL-1*β*), IL-6, cyclooxygenase-2 (COX-2), *β*-catenin, and Axin-2, which was mediated by the inactivation of the Wnt *β*-catenin signaling pathway [64, 67]. In another study, the survival rate of CRC cells, including cell proliferation, was evaluated to determine the underlying mechanisms for CUR's antiproliferative activity. SW480 and HCT116 cells were treated with CUR and exhibited the lowest viability, as well as loss of cell proliferation capabilities, which suggested that CUR inhibits cell proliferation of these cells by the inactivation of Wnt *β*-catenin signaling pathway. Specifically, it was suggested that the mechanism of inhibition of cell proliferation was Wnt *β*-catenin signaling-mediated overexpression of *β*-catenin, TCF4, microRNA-21 (miR-21), and miR-130a, in conjunction with downregulation of a negative regulator of this pathway (i.e., naked cuticle homolog 2 (Nkd2)) [68]. One of the most important microRNAs which is mostly downregulated in several cancers such as hepatocellular carcinoma and CRC is miR-491. This microRNA is also negatively associated with some of the mediators of Wnt *β*-catenin signaling including paternally expressed gene 10 (*PEG10*), *SIAH1* gene, Wnt1, Wnt3a, and *β*-catenin [69, 70]. Since cell proliferation of CRC cells is inhibited via CUR-mediated suppression of Wnt *β*-catenin signaling, this natural product may also potentially target miR-491/PEG10 and regulate cell proliferation during CRC recurrence. Accordingly, Li et al. reported that CUR reduced cell proliferation and stimulated apoptosis of HCT116 cell lines by upregulating *miR-491* and downregulating *PEG10* and *Wnt β-catenin* signaling [71].

In addition to CUR, its chemical derivatives, which include demethoxycurcumin (DMC), bisdemethoxycurcumin (BDMC), and tetrahydrocurcumin (THC), are also able to inhibit cell proliferation of various cancer cell lines via inactivation of Wnt *β*-catenin signaling [72, 73]. In line with these findings, another study investigated the potential of CUR and its derivates to inactivate Wnt *β*-catenin-induced aberrant cell proliferation of CRC cells. This study employed transfection of Wnt3a-conditioned medium (CM) treated HEK293 reporter (SEAP) cell lines into plasmid expressing hFz-1. Based on the activity of firefly luciferase (FL), it was demonstrated that CUR and its analogs inhibit cell proliferation of several CRC cell lines (e.g., HCT116, SW480, HCT15, and DLD-1) by decreasing FL and SEAP activity induced by Wnt3a-CM, reducing the expression and degradation of cytoplasmic *β*-catenin, and downregulating a positive regulator of this signaling pathway called *p-300* activator [74]. In contrast to the antiproliferative effects of CUR during CRC development, it has been shown that its derivative (THC) more effectively inhibits cell proliferation of various cancer cell lines when compared to CUR [75, 76]. Along these lines, analyses using IHC and western blotting in an AOM-induced colon carcinogenesis animal model showed that THC, in a concentration-dependent manner, could dramatically inhibit colonic epithelial cell proliferation when compared to CUR. These same authors proposed that THC's activity was achieved by reducing the expression levels of *β*-catenin, Wnt1, and GSK3*β*, which suggests that dietary consumption of THC may potentially decrease the number of aberrant crypt fuci (ACF) [77].

In addition to the antitumor effects of CUR in CRC, this natural compound can effectively modulate drug resistance of some chemotherapy drugs (e.g., 5-fluorouracil (5-FU)) during the development of the epithelial-mesenchymal transition (EMT) [78]. One of the most effective factors for increasing drug resistance in many cancers is EMT, which is associated with changes in cell polarity and the destruction of cell membranes and extracellular matrix [79]. In further support of CUR decreasing drug resistance of 5-FU during EMT, it has been demonstrated that following the addition of CUR to HCT116 cells, 5-FU-treated HCT116 cell lines show less proliferation, increased apoptosis, and suppression of the G0/G1 phase. These effects were suggested to occur due to a Wnt *β*-catenin signaling-mediated loss in the expression of tumor suppressor genes, including ten-eleven translocation1 (TET1) and NKD2, as well as an upregulation of *β-catenin*, *TCF4*, and *Axin* [80]. Importantly, as discussed below, CUR has also been evaluated in combination with other natural compounds to inhibit cancer cell proliferation.

Since CUR can potentially strengthen the inhibitory effects of 5-FU during EMT, it is possible that CUR may also synergistically augment the antitumor effects of the natural compound quercetin during EMT and thereby suppress the proliferation of CRC cells. Specifically, a previous study reported that the combination of CUR and quercetin synergistically inhibited the proliferation of HCT116 cells in a dose-dependent manner with an IC_50_ of 2.9 and 9.8 *μ*M, respectively [81]. Just as with the combination of CUR and quercetin, the pharmaceutical combination of CUR and salsalate (a nonsteroidal anti-inflammatory drug) can also potentially target several signaling pathways to suppress cell proliferation and colonic cytokines [58, 82]. Regarding the anti-inflammatory and anticancer effects of CUR and salsalate, these compounds can suppress Wnt *β*-catenin signaling-mediated colonic cell proliferation by decreasing the expression of colonic cytokines such as IL-6, IL-1*β*, and tumor necrosis factor-*α* (TNF-*α*), which are overexpressed in individuals with a high degree of adiposity secondary to high-fat diets. This suggests that overactivation of the Wnt *β*-catenin signaling pathway is accompanied by an increased number of inflammatory cytokines during tumorigenesis of colonic epithelial cells [83].

Biopolymers, such as micelles, have long been investigated to transport various drugs for site-specific drug therapy in an effort to improve drug targeting and efficacy, as well as minimize any side effects [84, 85]. In a previous study, two kinds of polymeric micelles, which included folate-polyethylene glycol (PEG)/hydrazone (Hyd) CUR/hydrophobic octadecylamine (C_18_)-*g*-polysuccinimide (PSI; FA-Cur) and PEG/Hyd-CUR/C_18_-*g*-PSI (NFA-Cur), were synthesized to improve the antitumor efficacy of CUR via Wnt *β*-catenin signaling-mediated cytoplasmic expression levels of *β*-catenin [86]. The findings demonstrated that both micelle forms could potentially suppress cell proliferation of SW480 CRC cell lines in a dose-dependent manner, although FA-Cur micelle treatment decreased the expression levels of *β*-catenin more effectively than NFA-Cur. Accordingly, these authors proposed that the mechanism responsible for the suppression of cell proliferation by both micellar formulations of CUR was due to a reduction in the expression of *β*-catenin in the cell cytoplasm and nucleus. They concluded that the anticancer effects of FA-Cur micelles were a promising candidate for colon cancer via the inhibition of the Wnt *β*-catenin signaling pathway [86].

### 2.5. Apoptosis and the Cell Cycle

In contrast to the antiproliferative activity of CUR in regulating the proliferation of CRC cells, it has been reported that CUR also increases apoptosis and reduces cell viability. It has been suggested that CUR mediates increased apoptosis and reduced cell viability by decreasing the expression levels of *β*-catenin nuclear translocation, Wnt3a, c-Myc, survivin, and cyclin D1. Additionally, when tested on SW620 human colonic adenocarcinoma cells, CUR exposure enhanced the expression of a negative regulator (i.e., caudal-type homeobox-2 (CDX2)) in the CDX2/Wnt *β*-catenin signaling pathway [87]. Another study by Jaiswal et al., which was conducted to determine the underlying mechanisms related to CUR's inhibition of CRC cell growth, demonstrated that CUR, in a time-dependent manner, caused p53- and p21-independent G(2)/M phase arrest and apoptosis in HCT116(p53^(+/+)^), HCT116(p53^(−/−)^), and HCT116(p21^(−/−)^) cell lines. This was suggested to occur due to an increase in caspase-3 expression-induced degradation of *β*-catenin and downregulation of c-Myc [88]. In addition to the apoptotic and cell cycle effects of CUR on CRC cells, it can also effectively reduce various cellular processes of cancer stem cells (CSCs). It is thought that CUR affects cellular processes of CSCs by targeting several signaling pathways that modulate the expression of select genes, which would seem to suggest that CUR may potentially target CSCs in many kinds of human cancers [89, 90]. Additionally, during the formation of CRC stem cells (CRC-SCs) and the development of increased drug resistance, it has been reported that CUR can also attenuate drug resistance to other chemotherapeutic drugs such as irinotecan (CPT-11), which is thought to be mediated by inhibition of several signaling pathways that affect the establishment and proliferation of CRC-SCs. Based on the positive relationship between CUR and CPT-11, as it pertains to drug resistance exhibited by CRC-SCs, another study demonstrated that CUR can significantly decrease drug resistance of LoVo-CPT-11 cells, inhibit features of CRC-SCs needed for sphere formation, and finally, stimulate apoptosis of sphere-forming cells. The effects of CUR on LoVo-CPT-11 cells were suggested to occur due to a reduction in the expression levels of various markers of CRC-SCs, which include the cluster of differentiation (CD) markers such as CD24, CD133, and CD44, as well as epithelial cell adhesion molecule (EpCAM) [91].

### 2.6. Metastasis and Invasion

In a study by Zhang et al. curcumin was evaluated for its potential to inhibit tumor EMT through the Wnt signaling pathway in colon cancer cells. Naked cuticle homolog 2 (Nkd2) small-interfering RNA (siRNA) and chemokine receptor 4 (CXCR4) expression plasmid were synthesized and transfected into curcumin-treated SW620 colorectal cancer cell lines, and the NKD2 and CXCR4 expression levels were determined. Their findings showed that curcumin significantly inhibited the proliferation of colorectal cancer cells and upregulated the expression of NKD2 in the cells, which resulted in the downregulation of key markers in Wnt signaling. Moreover, the progression of EMT was inhibited due to an overexpression of E-cadherin and downregulation of vimentin, as well as inhibition in tumor metastasis due to a significant downregulation in the expression of CXCR4 [92]. In addition to CUR's anticancer activity in metastasis and invasion of CRC cells, the combination of CUR with siRNAs to mediate restoration in the overexpression of select target genes may also potentially inhibit metastasis and invasiveness of CRC cells [93]. Along these lines, findings from a previous study demonstrated that combination of CUR with an siRNA targeting metastasis-associated lung adenocarcinoma transcript1 (si-MALAT1) can significantly suppress the migration and invasion of SW480 cells via downregulation in the expression levels of c-Myc, *ββ*-catenin, and cyclin D1, which is mediated by inactivation of the Wnt *ββ*-catenin cascade. This would suggest that si-MALAT1 can potentially increase the sensitivity of SW480 cells to CUR [93].

One of the most effective pathological processes for increasing the malignant nature of CRC is EMT, which plays a crucial role in promoting metastasis and invasion of human CRC cells [94]. To enhance metastasis and invasion of CRC cells during the EMT process, downstream mediators of the Wnt *ββ*-catenin pathway are essential [95, 96]. Curcumin can also potentially target components of Wnt *β*-catenin signaling, which include target genes, *β*-catenin, and tumor suppressors in an effort to suppress EMT-induced metastasis and invasion of CRC cells [97]. In one study, CUR was evaluated for its inhibitory effects on EMT in CRC cells. Human SW480 cells were incubated with increasing concentrations of CUR. DNA methylation levels of the CDX2 promoter were assessed, as were mRNA levels using RT-qPCR, protein expression levels using Western blotting, and nuclear translocation of *β*-catenin using immunofluorescence. Incubation of the cells with varying concentrations of CUR significantly downregulated the expression levels of DNA methytransferase1 (DNMT1) and the methylation levels of the CDX2 promoter in a concentration-dependent manner. In addition, the nuclear translocation levels of *β*-catenin were also reduced in a concentration-dependent manner. These authors concluded that CUR negatively regulated transcription factors promoting EMT in CRC cells by decreasing CDX2 promoter DNA methylation and consequently suppressing the CDX2/Wnt3a/*β*-catenin signaling pathway [97].

In contrast with these results, the anti-inflammatory effects of CUR were evaluated by Ahmed et al. in an animal model of colitis induced by *Citrobacter rodentium* infection and were shown to promote cell survival and mucosal regeneration by restoring Wnt *β*-catenin signaling. These investigators used a mouse model wherein a *C. rodentium* infection induced transmissible murine colonic hyperplasia (TMCH) and various degrees of inflammation. Chronic administration of dibenzazepine (DBZ) for 10 days was utilized to block both Notch and Wnt signaling, disrupt the intestinal barrier, and induce colitis. Dietary curcumin (4%) restored signaling via Notch and Wnt/*β*-catenin pathways, which promoted crypt regeneration, replenishment of the mucus layer, and thus, an amelioration of the *C. rodentium*- and DBZ-induced colitis. Therefore, CUR inhibited TMCH and colitis induced with DBZ administration and *C. rodentium* infection in mice [98] (Table 2). 

### 2.7. Anticancer Activity of Resveratrol Is Mediated by Wnt *β*-Catenin Signaling in Different Cellular Processes of CRC Cells

Based on the results of several previous studies, resveratrol can potentially target aberrant activation of several signaling pathways, including the Wnt *β*-catenin signaling pathway induced by various tumorigenic factors. Consequently, by inhibiting the Wnt *β*-catenin signaling pathway, resveratrol has been shown to affect several cellular processes of CRC cells such as cell proliferation, apoptosis, cell cycle dynamics, metastasis, and overall invasiveness.

### 2.8. Cell Proliferation

Resveratrol is known to inhibit cell proliferation and induce apoptosis in cancer cell lines at concentrations above 50 *μ*M. However, its effects on Wnt signaling are still being investigated. One study by Hope et al. examined the effects of RES on cell proliferation and Wnt signaling at low concentrations. Specifically, this study used two colon cancer cell lines (HT29 and RKO) and one normal mucosa-derived (NCM460) cell line. RES had no effect on cell proliferation at ≤40 *μ*M (HT29 and NCM460) and ˂20 *μ*M (RKO), although, at concentrations as low as 10 *μ*M, RES significantly decreased the amount and proportion of *β*-catenin in the nucleus of RKO and reduced the expression of *lgs* and *pygol* (regulators of *β*-catenin localization) in all cell lines. These authors concluded that at low concentrations in the absence of effects on cell proliferation, RES significantly inhibited Wnt signaling in colon-derived cancer cells, which appeared to be due in part to intracellular *β*-catenin localization [99]. Furthermore, RES can also target-specific signaling pathways involved in cell proliferation, which include the PI3K/Akt and Wnt *β*-catenin signaling pathways, to suppress cell proliferation of CRC cells [100]. In fact, it has been shown that RES significantly suppresses cell proliferation of HCT116 cells by an upregulation in the expression levels of phosphatase and tensin homolog (PTEN) and decreasing the phosphorylation of Akt1/2. The exogenous expression of PTEN inhibits the PIK/Akt signal and promotes the antiproliferative effects of RES in HCT116 cells, while knockdown of PTEN increases the PI3K/Akt signal but reduces the antiproliferative function of RES. Their findings also showed that the protein and mRNA expression of *β*-catenin are all decreased by RES in a concentration-dependent manner. Thus, these authors concluded that the antiproliferative effects of RES in human colon cancer cells may be mediated by regulating separately the PTEN/PI3K/Akt and Wnt *β*-catenin signaling [100].

Resveratrol has also been evaluated to determine whether it can target the *β*-catenin/T-cell factor (TCF) complex in the *β*-catenin signaling pathway. Using co-immunoprecipitation, together with *in vitro* binding assays, Chen et al. demonstrated that RES was capable of disrupting the binding between *β*-catenin and TCF4, which contributes to a decrease in Wnt signaling and Wnt *β*-catenin-mediated aberrant cell proliferation. Based on these findings, it was shown that RES is capable of identifying, and ultimately destroying, the *β*-catenin-TCF4 complex to inhibit the growth of P19 and COS-7 CRC cell lines mediated by inactivation of Wnt *β*-catenin. This process was suggested to be due to downregulation in the expression of Wnt target genes such as *cyclin D1*, *Axin2*, *ET-1*, and *c-Myc* without altering the expression levels of cytoplasmic and nuclear *β*-catenin [101]. In contrast to the antiproliferative role of RES in Wnt *β*-catenin pathway-mediated cell proliferation, it was demonstrated using Wnt signaling-specific microarray analysis and RT-qPCR that low doses of RES-containing freeze-dried grape powder (GP) did not significantly inhibit the Wnt pathway in colon cancer but had significant activity in inhibiting Wnt target gene expression in normal colonic mucosa obtained from eight patients. However, the GP, which contains low dosages of RES in combination with other bioactive components, at 80 g/day can inhibit the Wnt pathway *in vivo*, and this effect appeared to be confined to normal colonic mucosa [102].

Although RES's antiproliferative activity does not influence cell proliferation during CRC progression, it has been proven that RES can extensively target several mediators in CRC cells that are overexpressed (e.g., insulin growth factor1 (IGF-1)) and thereby suppress cell proliferation mediated by aberrant activation of Wnt *β*-catenin [103]. One of the most effective mediators for enhancing the antiproliferative effects of RES on the proliferation of CRC cells is IGF-1, which is highly expressed in the crypts of proliferating colonic cells during CRC progression [104]. In view of the significant negative relationship between RES and IGF-1, it has also been observed that RES can suppress cell proliferation of CRC cells by inhibiting IGF-1-induced aberrant activation of Akt/Wnt *β*-catenin signaling that mediates CRC cell growth [103]. Similar to IGF-1, prostaglandin E_2_ (PGE_2_) is highly expressed in CRC cells during tumorigenesis and is also considered a target for RES to effectuate inhibition of Wnt *β*-catenin signaling-mediated cell proliferation [105]. Indeed, it has been shown that RES can suppress cell proliferation and cell growth of PGE2-treated LS174T cells. It was proposed that the underlying mechanism responsible for this finding was inhibition of a PGE2-stimulated increase in downstream target genes of Wnt *β*-catenin, including the phosphorylation of GSK3*β*, the expression levels of cytoplasmic and nuclear *β*-catenin, and the expression levels of c-Myc and cyclin D1 [105].

### 2.9. Apoptosis and Cell Cycle

According to the findings of several recent studies, the antiproliferative and apoptotic effects of RES are mechanistically dependent on RES-bonded integrin *α*v*β*3-induced activation of extracellular signal-regulated kinase-1 and kinase-2 (ERK1/2) and stimulation of COX-2 accumulation. Accordingly, apoptosis of various human cancer cells is ultimately induced via upregulation of target genes (e.g., p53) involved in apoptosis [106, 107]. There are several strategies to enhance the antiproliferative and apoptotic activity of RES during CRC development. For example, it has been reported that the combination of RES with tetraiodothyroacetic acid (tetrac), as an anticancer agent, leads to a synergistic augmentation in the antiproliferative and apoptotic effects of RES. It is thought that that the combination of RES and tetrac to induce apoptosis of CRC cells is mediated by inhibiting Wnt *β*-catenin-induced overexpression of *β*-catenin and high mobility group protein A2 (HMGA2), as well as increasing both the nuclear accumulation of COX-2 and the expression levels of p53 [108]. In addition to RES promoting apoptosis mediated by ERK1/2 signaling, RES may also potentially target select regulators involved with Wnt *β*-catenin signaling disorder, such as IGF-1. This leads to suppression of cell proliferation and, ultimately, induces apoptosis of CRC cells. Along these lines, findings from a different study showed that following the addition of IGF-1 to HT-29 and SW480 cells, RES (100–150 *μ*M) exhibited antiproliferative properties in the HT-29 cells even after IGF-1 exposure by arresting the G_0_/G_1_-S phase of cell cycle progression via p27 stimulation and cyclin D1 suppression [109]. These authors concluded that RES inhibits CRC cell proliferation and elevates apoptosis (even in the presence of IGF-1) by suppressing the IGF-1/Akt/Wnt signaling pathway [74]. There are several effective factors, which include downstream target genes associated with Wnt *β*-catenin signaling (e.g., TCF-4 and c-Myc), that enhance the inhibitory effects of RES and induce RES-stimulated apoptosis [110]. In a study by Jeong et al., it was demonstrated that RES induced apoptosis of human CRC cell lines (including HCT116 and LoVo) in a dose and time-dependent manner by increasing the proteolytic proteasomal degradation of TCF4, reducing a downstream target of TCF4 (i.e., c-Myc), and decreasing exogenous Myc-tagged TCF4 without changing expression levels of *β*-catenin [110] (Table 3).

## 3. Conclusion

**Figure 1:**
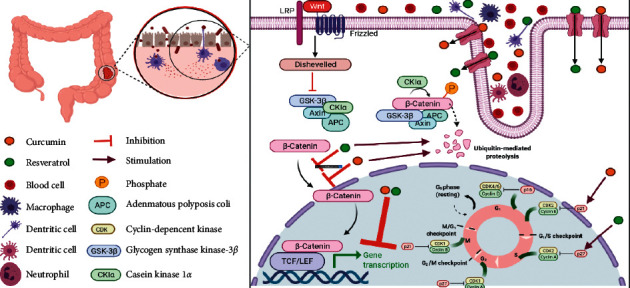
Schematic showing the therapeutic effects of CUR and RES on various types of colorectal cancer cell lines by regulation of transcription of genes involved in apoptosis and cell proliferation, which is mediated by Wnt *β*-catenin signaling.

In this review, we have summarized recent findings on the antitumor effects of CUR and RES in the pathogenesis of CRC as it pertains to the Wnt *β*-catenin signaling pathway (Figure 1). CUR and RES can target many of the mediators that regulate cellular processes of CRC cells via Wnt *β*-catenin signaling, suggesting that these natural compounds may represent appropriate therapeutic agents for the treatment of CRC. However, to consider both CUR and RES as suitable therapeutic interventions for the treatment of CRC, it is essential that the mechanism(s) underlying their effects to regulate both downstream and/or upstream mediators in signaling pathways associated with CRC progression and recurrence is (are) more clearly elucidated.

## Figures and Tables

**Table 1:** Therapeutic effects of some natural compounds on CRC in different stages of clinical trials.

Natural compounds	Interventions	Target	Phase	Dose	Duration	NCT number	Reference
Curcumin	Celecoxib	TCF/*β*-catenin	ǀǀǀ	N.M	N.M	NCT00295035	[56]
	5-Fluorouracil		ǀ	N.M	N.M	NCT02724202	[57]
	Irinotecan		ǀ	Oral curcumin (1,2,3, or 4 g/day) for 4 days + 200 mg/m^2^ irinotecan IV	June 2013–October 2016	NCT01859858	[58]

Resveratrol	SRT501	PDE4	ǀ	5.0 g oral administration of SRT501	August 2008–November 2009	NCT00920803	[54]

Genistein	mFOLFOX/mFOLFOX + Avastin	GSK3*β*	ǀ/ǀǀ	Combination genistein with mFOLFOX/mFOLFOX + Avastin (60 mg/day orally for 7 days every 2 weeks)	November 2013–October 2018	NCT01985763	—

Quercetin	Curcumin/rutin/quercetin/sulindac	—	Clinical	Oral sulindac (twice a day), oral rutin (1 of 3 doses twice a day), oral quercetin (1 of 3 doses twice a day), and oral curcumin (1 of 3 doses twice a day)	August 1996–July 2006	NCT00003365	—

Green tea	Fasting	—	Clinical	8 hours before CRC surgery and 8 hours after CRC surgery	January 2020–September 2023	NCT04345978	—
	GTE (green tea extract)	—	Clinical	0.9 g/day (GTE) and 0.6 g/day (EGCG)	August 2010–October 2015	NCT02321969	—

**Table 2:** Anticancer effects of curcumin on CRC cells via modulating Wnt *β*-catenin signaling.

Curcumin					
Effect	Action mechanism	Mediators	Combination therapy	Sample type	References
Antiproliferative	Suppressing AOM-DSS-induced cell proliferation	Reducing IL-1*β*, IL-6, COX-2, *β*-catenin, and Axin-2	—	Tissue (mouse model)	[64, 67]
	Inhibiting cell viability and cell proliferation	Inactivation of Wnt *β*-catenin along with decreasing expression levels of *β*-catenin, TCF4, miR-21, and miR-130a and upregulation of Nkd2	—	SW480 and HCT116	[68]
	Reducing cell proliferation	Inactivation of Wnt *β*-catenin-mediated downregulation of miR-491 and upregulation of PEG10	—	HCT116	[71]
	Inhibiting cell proliferation	Reducing FL and SEAP activity induced by Wnt3a-CM and downregulation of p-300	DMC, BDMC, and THC	HCT116, SW480, HCT15, and DLD1	[74]
	Decreasing cell proliferation, suppressing G0/G1 cell cycle, and inducing apoptosis	Inhibiting Wnt *β*-catenin; downregulation of *β*-catenin, TCF4, and Axin; and upregulation of TET1 and NKD2	5-FU	HCT116	[80]
	Suppressing colonic cell proliferation	Inactivation of Wnt *β*-catenin and reducing expression levels of IL-6, IL-1*β*, and TNF-*α*	SAL	Tissue	[83]
	Inhibiting cell proliferation	Suppression of *β*-catenin induced by Wnt3a-CM and LiCl-induced phosphorylation of GSK3*β*, stimulating transcriptional activity, and reducing *β*-catenin expression	FA-Cur and NFA-Cur	SW480	[86]

Apoptotic	Inducing apoptosis and reducing cell viability	Reducing expression levels of *β*-catenin, Wnt3a, c-Myc, survivin, and cyclin D1 as well as overexpression of CDX-2	—	SW620	[87]
	Suppressing G2/M cell cycle	Enhancing expression levels of caspase-3-induced degradation of *β*-catenin and downregulation of c-Myc	—	HCT116	[88]
	Inducing apoptosis of sphere-forming CRC-SCs, reducing drug resistance, and decreasing sphere formation	Reducing expression levels of CD44, CD133, CD24, and EpCAM	Irinotecan (CPT-11)	LoVo and CRC-SCs	[91]

Antimetastatic	Suppressing metastasis and invasion	Inhibiting Wnt *β*-catenin signaling; reducing expression levels of *β*-catenin, TCF4, Axin, and CXCR4; and increasing NKD2 expression	CXCR4 and NKD2	SW620	[92]
	Inhibiting metastasis and invasion	Inactivation of Wnt *β*-catenin signaling and downregulation of *β*-catenin, cyclin D1, and c-Myc	si-MALAT1	SW480	[93]
	Suppressing stimulation of EMT-induced metastasis and invasion	Inhibiting DNMT1, increasing expression levels of CDX-2, and decreasing *β*-catenin and Wnt3a expression	—	SW480	[97]

**Table 3:** Anticancer effects of resveratrol mediated by Wnt *β*-catenin signaling on CRC cells.

Resveratrol					
Effect	Action mechanism	Mediators	Targeted therapy	Sample type	Reference
Antiproliferative	Inhibiting cell proliferation	Inhibiting PI3K/Akt/Wnt *β*-catenin signaling, upregulation of PTEN, and decreasing *β*-catenin expression	PI3K/Akt/Wnt *β*-catenin signaling	HCT116	[100]
	Suppressing cell growth	Inactivation of Wnt *β*-catenin signaling and downregulation of cyclin D1, Axin2, ET-1, and c-Myc	TCF-*β*-catenin binding	P19 and COS-7	[101]
	Inhibiting cell growth and cell proliferation	Suppressing IGF-1	IGF-1	HT-29 and SW480	[103]
	Suppressing cell proliferation	Inhibiting PGE2 expression, downregulation of cyclin D1 and c-Myc, and decreasing GSK3*β* phosphorylation and *β*-catenin expression	PGE2	LS174T	[105]

Apoptotic	Inducing apoptosis	Increasing p53 and COX-2 expression and low-expression of *β*-catenin and HMGA2	Tetrac	HCT116 and HT-29	[108]
	Stimulating apoptosis and inhibiting cell proliferation	Upregulation of p27 and p53, arresting G0/G1-S cell cycle phase, and low expression of cyclin D1	IGF-1	HT-29 and SW480	[109]
	Increasing apoptosis	Enhancing proteolytic proteasomal degradation induced by TCF4, decreasing TCF4 and c-Myc expression, as well as reducing exogenous myc-tagged TCF4	TCF-4 and c-Myc	LoVo and HCT116	[110]

## Data Availability

No raw data were associated with this review.

## Abbreviations

CRC: Colorectal cancer
CUR: Curcumin
RES: Resveratrol
Wnt: Wingless transduction
APC: Adenomatous polyposis coli
GSK3*β*: Glycogen synthase kinase 3*β*
CK1*α*: Casein kinase 1*α*
*β*-TrCP: *β*-Transducin repeat-containing protein
Fzd: Frizzled receptor
LRP5/6: Lipoprotein receptor-related protein 5/6
Dvl: Disheveled
TCF/LEF: T-cell factor/lymphocyte enhancer factor
AOM: Azoxymethane
DSS: Dextran sodium sulfate
DBZ: Dibenzazepine
RT-qPCR: Real-time quantitative polymerase chain reaction
IHC: Immunohistochemistry
IL-1*β*: Interleukin-1*β*
COX-2: Cyclooxygenase-2
miR-21: MicroRNA-21
Nkd-2: Naked cuticle homolog 2
PEG 10: Paternally expressed gene 10
DMC: Demethoxycurcumin
BDMC: Bisdemethoxycurcumin
THC: Tetrahydrocurcumin
SEAP: Wnt3a-conditioned medium (CM) treated HEK293 reporter
FL: Firefly luciferase
ACF: Aberrant crypt fuci
5-FU: 5-Fluorouracil
EMT: Epithelial-mesenchymal transition
TET-1: Ten-eleven translocation1
TNF-*α*: Tumor necrosis factor-*α*
PEG: Polyethylene glycol
Hyd: Hydrazone
PSI: Polysuccinimide
CDX-2: Caudal-type homeobox-2
CSC: Cancer stem cell
CRC-SC: Colorectal cancer stem cell
CPT-11: Irinotecan
CD: Cluster of differentiation
EpCAM: Epithelial cell adhesion molecule
siRNA: Small-interfering RNA
CXCR4: Chemokine receptor 4
Si-MALAT1: siRNA targeting metastasis-associated lung adenocarcinoma transcript1
DNMT1: DNA methytransferase1
PTEN: Phosphatase and tensin homolog
IGF-1: Insulin growth factor1
PGE2: Prostaglandin E_2_
ERK1/2: Extracellular signal-regulated kinase-1 and kinase-2.
